# Targeting Human MicroRNA Genes Using Engineered Tal-Effector Nucleases (TALENs)

**DOI:** 10.1371/journal.pone.0063074

**Published:** 2013-05-07

**Authors:** Ruozhen Hu, Jared Wallace, Timothy J. Dahlem, David Jonah Grunwald, Ryan M. O'Connell

**Affiliations:** 1 Department of Pathology, University of Utah, Salt Lake City, Utah, United States of America; 2 Department of Human Genetics, University of Utah, Salt Lake City, Utah, United States of America; Cincinnati Children's Hospital Medical Center, United States of America

## Abstract

MicroRNAs (miRNAs) have quickly emerged as important regulators of mammalian physiology owing to their precise control over the expression of critical protein coding genes. Despite significant progress in our understanding of how miRNAs function in mice, there remains a fundamental need to be able to target and edit miRNA genes in the human genome. Here, we report a novel approach to disrupting human miRNA genes *ex vivo* using engineered TAL-effector (TALE) proteins to function as nucleases (TALENs) that specifically target and disrupt human miRNA genes. We demonstrate that functional TALEN pairs can be designed to enable disruption of miRNA seed regions, or removal of entire hairpin sequences, and use this approach to successfully target several physiologically relevant human miRNAs including miR-155*, miR-155, miR-146a and miR-125b. This technology will allow for a substantially improved capacity to study the regulation and function of miRNAs in human cells, and could be developed into a strategic means by which miRNAs can be targeted therapeutically during human disease.

## Introduction

MicroRNAs are small, single-stranded RNAs that have been highly conserved during evolution and function by repressing target gene expression. miRNAs have recently emerged as critical modulators of gene expression networks in mammals, and their impaired expression or function has been linked to a variety of human diseases [1]. A wide range of human cancer cell types display dysregulated miRNA expression patterns, and there is overwhelming evidence that some miRNAs are functionally relevant in malignancies by playing imperative tumor suppressor roles or by acting as aggressive oncogenes. In addition to cancer, miRNAs are also perturbed in cardiovascular disease [2], neurological disorders [3], and autoimmunity [1], [4], and this is consistent with miRNAs having obligatory regulatory roles in a variety of human organ systems. Beyond disease, miRNAs can also participate in the formation of induced pluripotent stem (iPS) cells [5], which hold significant promise in the field of regenerative medicine.

Despite these important and clinically significant roles for miRNAs, our ability to manipulate miRNA expression and function in human cells remains a challenging task. For instance, unlike protein coding mRNAs, siRNAs cannot be used to reduce miRNA levels within cells. Delivery of oligonucleotides antisense to target miRNAs has had some success, but is limited to certain cell types that can uptake these oligonucleotides with high efficiency, such as hepatocytes, and requires constant delivery of fresh inhibitor [6]. Thus, novel approaches to regulating miRNA expression or function in human cells are clearly needed and should have a substantial impact on our ability to study human physiology, combat human diseases, and regenerate damaged tissues. Following their transcription in the cell nucleus, miRNAs undergo a series of processing steps before reaching maturity [7]. The fully processed miRNA is loaded into RISC and then mediates target gene repression by directing the RISC complex to specific mRNA 3′ UTRs containing cognate binding sites for the miRNA. This interaction between the miRNA and 3′ UTR is dependent upon a 6–8 nucleotide sequence found in the 5′ end of the miRNA called the “seed” sequence. This sequence must have perfect complementarity with its 3′ UTR binding site for repression to occur. Disruption of the seed region of the miRNA or cognate binding site abolishes repression and thus miRNA function. Therefore, miRNAs can be modulated by controlling their expression levels or by disrupting their seed:target interaction.

Recently, a novel class of DNA-binding proteins from *Xanthomonas* plant pathogens, called Transcription Activator-Like Effectors (TALEs), have been shown to bind DNA in a highly sequence specific manner and mediate gene modifications based upon their fusion to trans-activation, repression or nuclease domains [8]. Importantly, because TALE proteins are made up of modules, with each interchangeable module recognizing specific DNA bases, TALEs can theoretically be engineered to bind virtually any DNA sequence. Just recently TALE proteins have been shown to function in human cells indicating that this technology can be used to modify specific human genes [9], [10].

In the present study, we have developed custom TALENs that have been engineered to target 4 specific miRNAs with established functional importance, and these include miR-155, miR-155*, miR-146a and miR-125b. We demonstrate that in all cases we can achieve sequence deletions within these genes that include disruptions to the miRNA seed sequence, and achieve complete miR-155 hairpin removal by using two TALEN pairs together. Furthermore, we observe bi-allelic modifications indicating that TALENs can disrupt both miRNA gene alleles within a human cell. This work describes a novel approach to targeting and disrupting miRNA genes in the human genome, and has important implications for both basic and translational research involving miRNAs.

## Results

### Design and construction of miRNA-targeting TALENs

**Figure 1:**
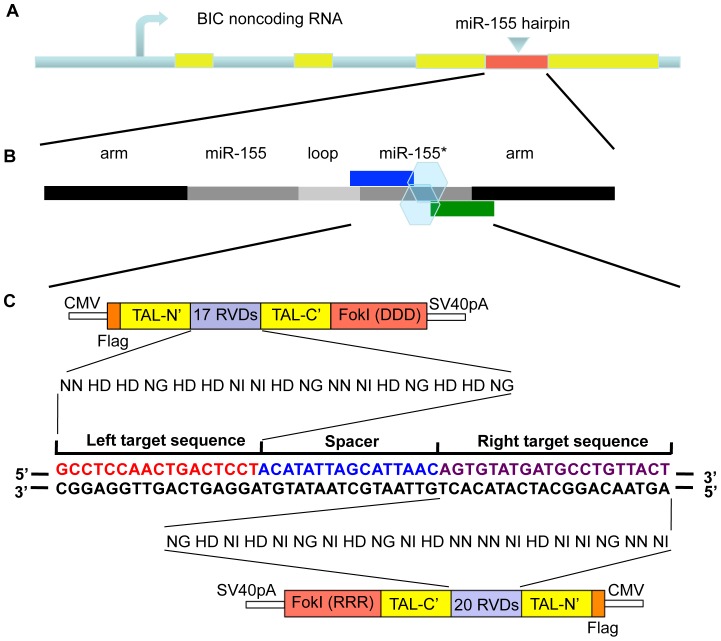
Schematic of the miR-155/miR-155* genomic locus and the location of the TALEN pair engineered to target the miR-155* region. (A) Schematic of the miR-155/miR-155* genomic locus. The three BIC exons are shown in yellow and the miR-155 hairpin is in red. (B) Schematic of the miR-155 hairpin structure. The miR-155 arms are shown in black, while the mature miR-155 and miR-155* sequences are in dark grey. Blue and green boxes represent the binding sites of the TALEN pair designed to target the miR-155* region, and hexagons represent the heterodimerized FokI enzyme positioned over the spacer sequence. (C) The two expression plasmids containing the TALEN pair along with the FokI nuclease domain, and the TALEN-RVD sequences corresponding to each targeted DNA sequence are shown. The details of pCS2TAL3-DDD and pCS2TAL3-RRR expression vectors are described in the Materials and Methods section. NN, HD, NG and NI represent the RVD regions of each repeat sequence that bind to nucleotide G, C, T and A, respectively. The left and right TALEN binding sequences are shown in red and purple, respectively, and the spacer region is in blue.

To develop TALE proteins with the capacity to modify specific miRNA gene sequences, we first used an *in silico* approach to identify promising TALE protein pair binding sites that flank the sequences of a specific human miRNA of interest, miR-155*. Our analysis was carried out using TALE-NT software (https://boglab.plp.iastate.edu/node/add/talen), and followed the parameters described in our Materials and Methods. This led to the identification of a putative TALE binding site flanking the miR-155* sequence. Next, TALE-repeat variable dinucleotides (RVDs) corresponding to the specific target DNA sequences were cloned into expression plasmids using the Golden Gate assembly system as described in the Materials and Methods section. The expression plasmids were comprised of the TALE DNA binding modules, a short linker, and a modified FokI nuclease domain (Fig. 1). To increase target specificity, we used modified FokI nuclease domains that function as obligate heterodimers. Consequently, both the ‘Left’ and ‘Right’ TALE nuclease (TALEN) proteins that make up the pair must bind simultaneously to their DNA sites in the genome for Fok1 to heterodimerize and become an active enzyme. With this design, the DNA spacer sequence between the bound TALENs is cut, and deletions or other mutations are introduced during non-homologous end joining (NHEJ) DNA repair of the cut DNA.

### The miR-155* targeting TALEN pair facilitates deletions in the miR-155* sequence

**Figure 2:**
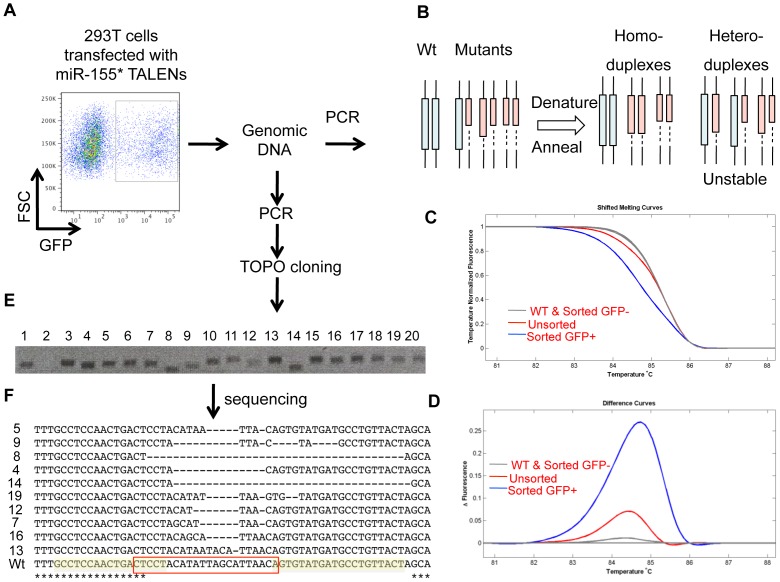
The miR-155* targeting TALEN pair causes mutations in the miR-155* sequence. 293T cells were transfected with or without plasmids encoding the TALEN pair designed to target the miR-155* region. A GFP expressing plasmid was co-transfected. (A) 48 hours later, GFP+ cells were sorted by FACS and subjected to gDNA extraction. (B–F) The miR-155* targeted region was amplified by PCR and subjected to an HRMA analysis (B–D) or TOPO cloning and sequencing (E,F). (B) Schematic of the HRMA approach. A small region of the genome that includes different lengths of DNA deletions is amplified by PCR. Upon annealing, different types of homoduplex and heteroduplex dsDNA molecules are produced with different melting temperatures. (C) HRMA of the mir-155* PCR amplicons generated using gDNA from Wt (mock transfected 293t cells), Unsorted (293t cells with the TALEN pair transfection), Sorted GFP+ and sorted GFP-(293t cells with the TALEN pair and a GFP plasmid co-transfection followed by FACS sorting). (D) The results of the HRMA analysis are also shown as fluorescence difference plots using the normalized data. The Wt sample is used as the baseline. (E) PCR products from C were cloned into a TOPO vector and the length of the individual DNA fragment was assessed by gel electrophoresis. (F) Sequencing results of TOPO clones from E are shown. They are aligned with the wild-type miR-155* sequence. The left and right TALEN binding sites are highlighted in yellow and the miR-155* region is boxed in red.

After construction of the miR-155* TALEN pair (TALEN A) expression plasmids, we subjected this TALEN pair to a functional analysis pipeline to assess its ability to target and mutate the intended sequence in the human genome (Fig. S1). Equal amounts of the miR-155* TALEN pair expressing plasmids, along with a GFP expressing vector, were transfected into human HEK 293T cells. 48 hours later, GFP+ cells were isolated using FACS (Fig. 2A) and the gDNA was extracted from the cells and analyzed for the presence of mutations to the desired miRNA gene region.

To initially screen for the presence of DNA sequence modifications, short PCR amplicons (90–150 bp) that included the region of interest were generated from the gDNA samples (Fig. 2A,B). The PCR product was next subjected to a High Resolution Melt Analysis (HRMA) analysis, described previously [11]. If TALEN-induced mutations were present in the template gDNA, the thermostability of the dsDNA population of renatured PCR amplicons would be different from amplicons produced using wild-type gDNA samples (Fig. 2B). Consistent with this, amplicons from gDNA taken from the miR-155* TALEN A pair transfected cells had an altered thermostability compared to control cells not receiving the TALEN pair. Furthermore, the difference was more significant when we compared the sorted GFP+ cells with unsorted cells, while there were no thermostability differences between Wt cells and sorted GFP- cells from the TALEN transfected cell culture (Fig. 2C). Upon generating fluorescence difference plots, we found that the curves for the Wt and sorted GFP- samples were clustered around the baseline while the curves for the TALEN transfected samples (GFP+) were clearly above background (Fig. 2D). These data indicated that the miR-155* TALEN A pair caused sequence modifications, potentially within the desired region.

The PCR products containing TALEN-targeted mutations were next subjected to TOPO cloning. Individual TOPO clones containing single PCR DNA fragments were analyzed by gel electrophoresis or sequenced (Fig. 2E,F). Several clones were found to be of different sizes, indicating the presence of deletions within the targeted genomic region (Fig. 2E). The relative sizes of the deletions were also consistent with sequencing data, which confirmed the presence of deletions of varying lengths (Fig. 2F). These findings demonstrate that the miR-155* TALEN A pair causes targeted deletions in the mature human miR-155* sequence. Although these deletions are specific to the miR-155* sequence, they will also disrupt the stem-loop structure containing miR-155 and miR-155*. This is expected to inhibit processing and production of both mature miR-155 and miR-155*.

### The miR-155* TALEN pair elicits both bi-allelic and mono-allelic mutations

**Figure 3:**
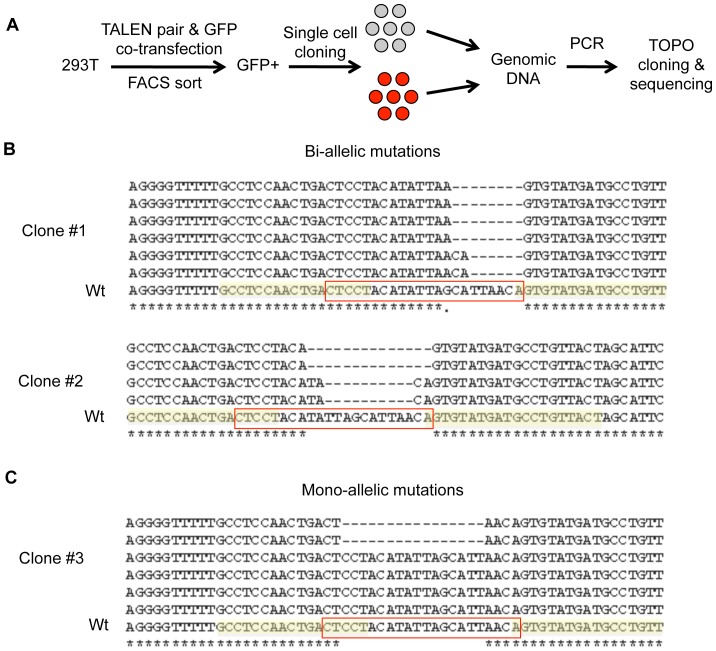
The miR-155* targeting TALEN pair causes both bi-allelic and mono-allelic mutations in human cells. (A) Schematic of the experimental design. 293T cells were transfected with the TALEN pair targeting the miR-155* region along with a GFP plasmid. 48 hours later, GFP+ cells were sorted by FACS and subjected to single cell cloning. After individual cell clones were expanded, gDNA was extracted from the cell clones. The miR-155* TALEN pair-targeted region was amplified by PCR and subjected to TOPO cloning and sequencing. (B,C) Representative cell clones showing bi-allelic mutations (B) or mono-allelic mutations (C). In the sequence alignment graph, the left and right TALEN binding sites are highlighted in yellow and the miR-155* region is in the red box.

Although able to successfully target the miR-155* sequence using our TALEN A pair, we next assessed if mono- or bi-allelic modifications were occurring (Fig. 2B). Because each cell has two alleles of an individual miRNA gene, it was important to determine if we could disrupt both copies, which would completely abolish the function of the target miRNA. To make this determination, FACS-sorted TALEN-transfected GFP+ cells were used to generate clonal populations (Fig. 3A). gDNA was extracted from clonal populations and subjected to PCR to amplify the targeted sequence. The amplicons from 3 different cell clones with TALEN-mediated deletions were then subjected to TOPO cloning followed by sequencing to decipher the precise mutations that had occurred in the targeted region in each clonal population. The sequencing results revealed that some cell clones had two unique deletions and no Wt alleles, consistent with a bi-allelic alteration, while other clones had one Wt allele and a deleted sequence, consistent with a mono-allelic modification (Fig. 3B,C). These data indicate that bi-allelic modifications to miRNA genes can be mediated by TALENs.

### Complete deletion of the miR-155 hairpin by using two TALEN pairs together

**Figure 4:**
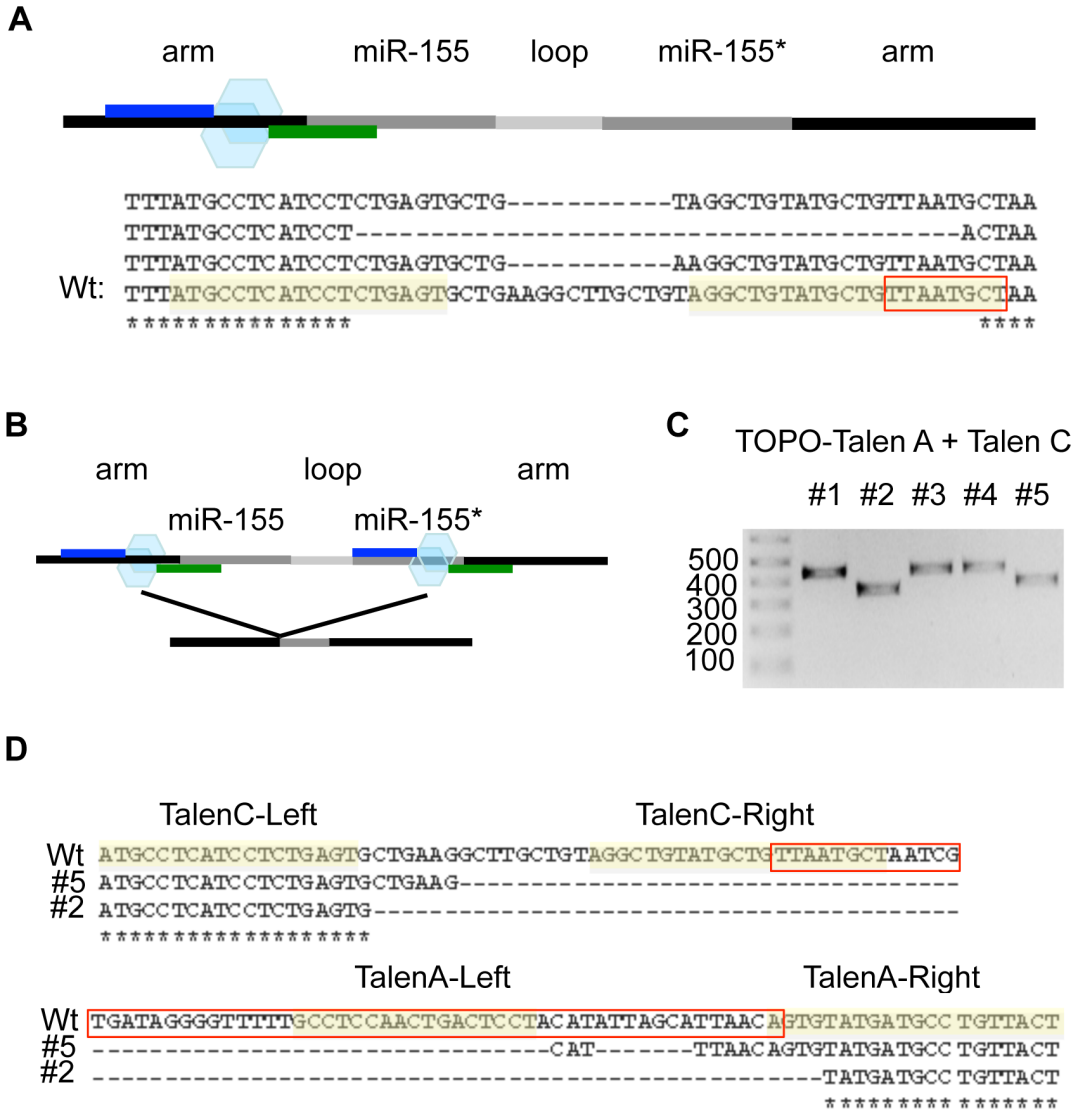
Using two TALEN pairs to delete the entire human miR-155 hairpin sequence. (A) TALEN pairs targeting miR-155 were designed and constructed (called TALEN C). The upper panel shows a schematic of the TALEN A pair binding sites. The lower panel shows the sequence alignments comparing Wt and TALEN C mutated miR-155. The left and right TALEN binding sites are highlighted in yellow and the miR-155 seed region is boxed in red. (B) Schematic of the binding sites of two TALEN pairs (TALEN A and TALEN C) targeting miR-155. (C–D) Both TALEN A and TALEN C pairs were transfected into 293T cells. The miR-155 locus was amplified by PCR and subjected to TOPO cloning and sequencing. (C) Electrophoresis gel analysis showing deletions in the miR-155 locus. The arrows on the right indicate the two expected PCR products with or without large deletions. (D) Sequence alignments between a Wt clone and two TOPO clones with large deletions. The left and right TALEN binding sites for both TALEN A and TALEN C are highlighted in yellow and the miR-155 hairpin sequence is boxed in red.

In order to target miR-155, we designed another TALEN pair (TALEN C) that bind just upstream from the 8-nucleotide miR-155 seed region. Sequencing showed that the miR-155 TALEN C caused deletions in the desired locus that included the miR-155 seed region in some cases (Fig. 4A).

Since both miR-155 TALEN pairs A and C target each end of the miR-155 hairpin sequence, we tested whether the combination of these two TALEN pairs could delete the entire sequence that constitutes pre-miR-155 (Fig. 4B). Both TALEN A and C pairs were co-transfected into 293T cells and gDNA was analyzed by PCR and TOPO cloning as described in Fig. 2. Using gel electrophoresis we found that several clones were approximately 80 bps smaller than the Wt sequence, indicating the presence of larger deletions within the targeted miR-155 locus (Fig. 4C). Sequencing data confirmed the presence of deletions that span the entire miR-155 hairpin sequence (Fig. 4D). These findings demonstrate that the combination of the two TALEN pairs caused complete deletion of human pre-miR-155.

### Targeting of human miR-146a and miR-125b1 using engineered TALENs

**Figure 5:**
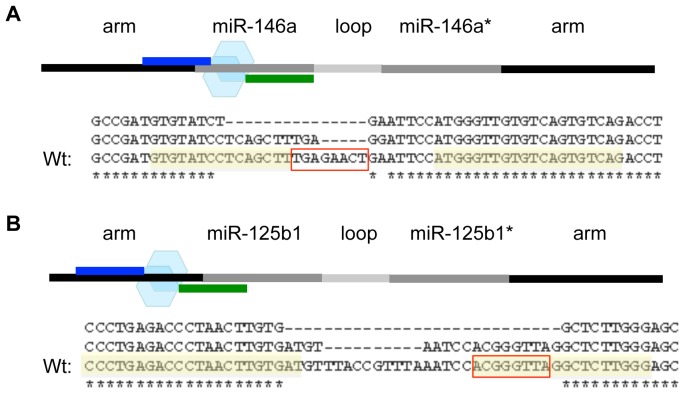
Using TALENs to target the seeds of human miR-146a and miR-125b1. TALEN pairs targeting the indicated miRNAs were designed and constructed. After the TALEN pairs were transfected into 293T cells, methods were performed (as described in Figure 2) to detect mutations in the targeted regions. (A, B) The upper panel shows the schematics of the TALEN pair binding sites adjacent to (A) miR-146a and (B) miR-125b1. The lower panel shows the sequence alignments between the Wt and mutated miRNA genes. The left and right TALEN binding sites are highlighted in yellow and the miRNA seed regions are boxed in red.

We next used this same overall approach to design, build and test 2 other TALEN pairs against additional human miRNAs of interest, including miR-146a and miR-125b1. For miR-125b1, we designed the TALENs to target sequences just upstream instead of flanking the miRNA seed region, and did so to stay within the design parameters. However, for miR-146a, we identified promising TALEN sites that flanked its seed sequence (Fig. 5A). Like the miR-155* and miR-155 TALENs, each new TALEN pair successfully targeted and mutated the expected miRNA gene sequences, albeit at different efficiencies (Fig. 5A,B and Table 1). Interestingly, all of the TALEN pairs caused at least some deletions that disrupted the seed sequences of each respective miRNA, even if their binding sites did not flank, but were near, the seed. These results indicate that TALENs can be routinely used to disrupt human miRNA seed sequences, and that the design parameters consistently allow for successful targeting of a DNA sequence as small as an 8-nucleotide miRNA seed found in specific DNA locations and contexts.

**Table 1:** Mutation rate mediated by TALEN pairs in transfected 293T cells.

Talen targeting miRNA	Total Topo clones sequenced	Topo clones with mutations	Mutation rate
miR-155* Talen A	19	10	52.6%
miR-155 Talen C	12	5	41.7%
miR-146a	34	5	14.7%
miR-125b1	18	2	11.1%

## Discussion

Our study set out to determine whether TALEN technology could be used to target miRNA genes in human cells. Unlike protein coding genes that are typically made up of thousands of nucleotides from which optimal TALEN binding sites can be found, relevant miRNA gene sequences are considerably smaller, which limits the likelihood of finding well positioned TALEN sites. However, our results indicate that TALENs can be repeatedly designed to target specific miRNA loci and achieve miRNA seed disruption, or miRNA hairpin removal when two TALEN pairs are used together. Because miRNA-mediated gene repression is dependent upon its seed sequence, this approach can be used to permanently block the function of specific human miRNAs.

Although our TALENs successfully disrupted miRNA gene seeds, the resulting deletions were heterogeneous in nature as reported by others [9], [10]. Thus, although one can disrupt miRNA function using this method, the resulting modification is variable. It has recently been demonstrated that DNA editing can be achieved using TALENs and a single-stranded donor DNA molecule with homologous arms [12]. Future work should use this method to edit miRNA seed sequences in a manner that prohibits, or alters, their targeting capacity or specificity in a controlled manner. Furthermore, such an editing approach could also be used to modify miRNA-binding sites in the 3′ UTRs of specific target genes, or polymorphisms within *cis* regulatory elements that influence miRNA expression. A recent example of such a polymorphism is found in miR-146a, which has a G/C polymorphism within in pre-miRNA sequence that reduces its expression and contributes to a predisposition to papillary thyroid cancer [13]. Polymorphisms within the miR-155 gene have also been associated with its altered expression in human Multiple Sclerosis patients [14].

We also found that each TALEN pair had a different functional efficiency as determined by the rate of target allele mutations (Table 1). This indicates that despite following established design guidelines, additional factors are able to influence TALEN function. These may include chromatin structure, DNA modifications such as methylation, or other DNA sequence variations that influence TALEN binding dynamics. However, such determinants are presently being investigated, as this is a relatively new field of study. The capacity to deliver TALENs to precise cell types is also a challenging endeavor. Similar to other studies, we have demonstrated that transfection of cells with plasmids encoding the TALEN pair can be used to express TALENs in target cells [10]. However, the development of viral vector systems that enable transient expression of TALENs in specific cell types is necessary for many important applications *in vivo* [15].

As we continue to understand how miRNAs regulate mammalian biology, both in physiological and pathological contexts, it is becoming increasingly necessary to develop tools with the ability to specifically target and modify human miRNA genes *in vivo*. Based upon our findings here, TALENs make excellent candidates to achieve miRNA gene targeting and manipulation in a variety of relevant human cell types, including those with important therapeutic applications, such as stem cells, neurons and primary tumors.

## Materials and Methods

### Cell culture and transfection

HEK 293T cells were obtained from the American Type Culture Collection (Rockland, MD) and were cultured with DMEM supplemented with antibiotics and 10% FBS. Cells were maintained at 37°C in a humidified incubator supplied with 5% CO_2_. For transfection of plasmids, TransIt-293 transfection reagent (Mirus, WI) was used to transfect 293T cells according to the manufacturer's protocol. The TALENs were transfected at a molar ratio of 1∶1.

### TALEN target site design

TALEN target sites were designed as described in Dahlem et al [11]. Briefly, the TALEN Targeter (old version) program at https://boglab.plp.iastate.edu/node/add/talen was used to scan the sequences flanking the miRNAs of interest (including miR-155*, miR-155, miR-146a and miR-125b1) for potential TALEN pair target sites. Site selection was restricted using the following parameters: 1) spacer length between TALENs: 14–17; 2) TALE repeat array length of 16–21 and 3) by applying all additional options that restrict target choice. Preference was given to target sites where the spacer centered on or near the seed regions of the miRNAs and therefore would likely induce loss of function deletions. The uniqueness of potential TALEN target sequences was determined using the Target Finder (https://boglab.plp.iastate.edu/node/add/talen) and a BLAST analysis, ensuring that highly similar Left and Right binding sites in close proximity did not exist at other regions in the human genome. The designed TALEN pair and spacer sequences are as follows: miR-155*-Talen A-left: GCCTCCAACTGACTCCT; miR-155*-Talen A-right: AGTGTATGATGCCTGTTACT; miR-155*-Talen A-spacer: ACATATTAGCATTAAC; miR-155-Talen C-left: ATGCCTCATCCTCTGAGT; miR-155-Talen C-right: AGGCTGTATGCTGTTAATGCT; miR-155-Talen C-spacer: GCTGAAGGCTTGCTGT; miR-146a-Talen-left: GTGTATCCTCAGCTT; miR-146a-Talen-right: ATGGGTTGTGTCAGTGTCAG; miR-146a-Talen-spacer: TGAGAACTGAATTCC; miR-125b1-Talen-left: CCCTGAGACCCTAACTTGTGAT; miR-125b1-Talen-right: ACGGGTTAGGCTCTTGGG; miR-125b1-Talen-spacer: GTTTACCGTTTAAATCC.

### TALEN assembly and expression plasmid construction

TALEN pairs were designed and constructed by the Mutation Generation and Detection Facility at the University of Utah (http://www.cores.utah.edu/). The TALEN Golden Gate kit described by Cermark et al [9] was used and TALENs were assembled as described by Dahlem et al [11]. Each nucleotide of a target site is recognized by one repeat module of the TALEN protein. Two amino acids within each module, called the Repeat Variable Di-residues (RVDs), are responsible for nucleotide recognition. RVDs NI, NN, NG, and HD bind to nucleotides A, G, T, and C, respectively. The TALEN Golden Gate Kit contains plasmids containing each of the individual RVD modules along with intermediate cloning plasmids and a final expression vector. These reagents were used to construct specific TALEN expression vectors. The TALEN Golden Gate Kit (#1000000024) was obtained from Addgene. Briefly, successive rounds of Golden Gate cloning assembly were used to generate TALEN expressing plasmids with n RVD repeat modules. First, two arrays corresponding to repeat modules 1–10 and 11-n-1 were assembled into separate intermediate vectors and those acquiring RVD arrays were screened on IPTG/X-gal plates. Correct assembly was determined first by XbaI and AflII restriction enzyme digestion followed by sequencing of plasmids with the correct insert sizes. Second, the two arrays and sequences encoding the n^th^ motif were assembled into the final expression backbone vectors pCS2TAL3-DDD and pCS2TAL3-RRR to generate a left and right TALEN expressing plasmids, respectively, followed by screening on IPTG/X-gal plates. Correct assembly was determined first by SphI and BamHI restriction enzyme digestion followed by sequencing of plasmids with the correct insert sizes.

The pCS2TAL3-DDD and pCS2TAL3-RRR final expression vectors were modified from pCS2TAL3-DD and pCS2TAL3-RR plasmids described in Dahlem et al [11] with extra mutations in the FoKI nuclease domain. In pCS2TAL3-DDD the amino acid change of H496D was introduced into the Fok I domain and in pCS2TAL3-RRR the amino acid change H537R was introduced [16], [17]. The DDD and RRR mutations within the Fok I nuclease domains require that the TALENs function as obligate heterodimers, requiring both ‘Left’ and ‘Right’ monomers to simultaneously recognize their cognate binding sites to achieve nuclease activity. The pCS2TAL3 final backbone vectors contain the simian IE94 cytomegalovirus eukaryotic enhancer/promoter (CMV), the recognition sequence used by the prokaryotic SP6 RNA polymerase (SP6), and the polyadenylation signal sequence derived from SV40 (SV40pA) taken from the pCS2+ plasmid (http://sitemaker.umich.edu/dlturner.vect ors). Other domains include a nuclear localization signal (NLS); the FLAG epitope (Flag); and truncated TAL protein N-terminus and C-terminus (TAL-N′ and TAL-C′) sequences derived from pTAL3 [9].

### Genomic DNA extraction

Genomic (g) DNA was extracted from transfected 293T cells or single cell clones by using DNeasy Blood & Tissue Kit (QIAgen, MD). The average gDNA concentration was adjusted to 30 ng/ul for PCR reactions.

### High Resolution Melt Analysis (HRMA)

To detect TALEN-induced mutations by HRMA, a ∼100–150 bp amplicon that included the entire genomic target site was amplified by PCR. Primers flanking the target site were used to amplify the genomic region in a 10 ul PCR reaction containing: 1 ul gDNA (30 ng/µl), 1× LightScanner Master Mix (containing the LC Green Plus dye, Idaho Technology), 200 µM dNTP, and 200 nM each Forward and Reverse primers. Amplification/duplex formation conditions were: 94°C, 3 min; 50 cycles [94°C, 30 s; 70°C, 17 s]; 94°C, 30 s; 25°C, 30 s; 10°C. HRMA data was collected on a LightScanner (Idaho Technology) and analyzed using LightScanner Call-IT Software. The primer sequences are available upon request.

### TALEN-induced mutation screening

Amplicons were cloned using the TOPO TA Cloning Kit (Invitrogen). TOPO plasmids were digested and the inserted PCR DNA fragments were analyzed by gel electrophoresis. In parallel, TOPO plasmids containing the amplified DNA clones were sequenced at the DNA sequencing core facility at University of Utah.

## Supporting Information

Figure S1 **Experimental plan used to develop miRNA-targeting TALENs.** TALEN pairs targeting different miRNAs were designed, constructed and transfected into 293T cells along with a GFP expression vector. After transfection, 293T cells were subjected to FACS sort to isolate cells with the TALEN pairs. gDNA was extracted from Wt, unsorted, GFP- or GFP+ cells. The TALEN pair-targeted regions were amplified by PCR and subjected to HRMA or TOPO cloning and sequencing to determine the presence of mutations. Moreover, GFP+ cells were plated in 96 well plates to obtain single cell clones. Single cell clones were subjected to PCR and TOPO cloning analyses to determine if bi- or mono-allelic mutations were being generated within the TALEN targeted regions.
(TIF)
