# Supplementary figures and images for: Targeting Human MicroRNA Genes Using Engineered Tal-Effector Nucleases (TALENs)
Source: PLoS One. 2013 May 7;8(5):e63074. doi: 10.1371/journal.pone.0063074 (PMC3646762; doi:10.1371/journal.pone.0063074)

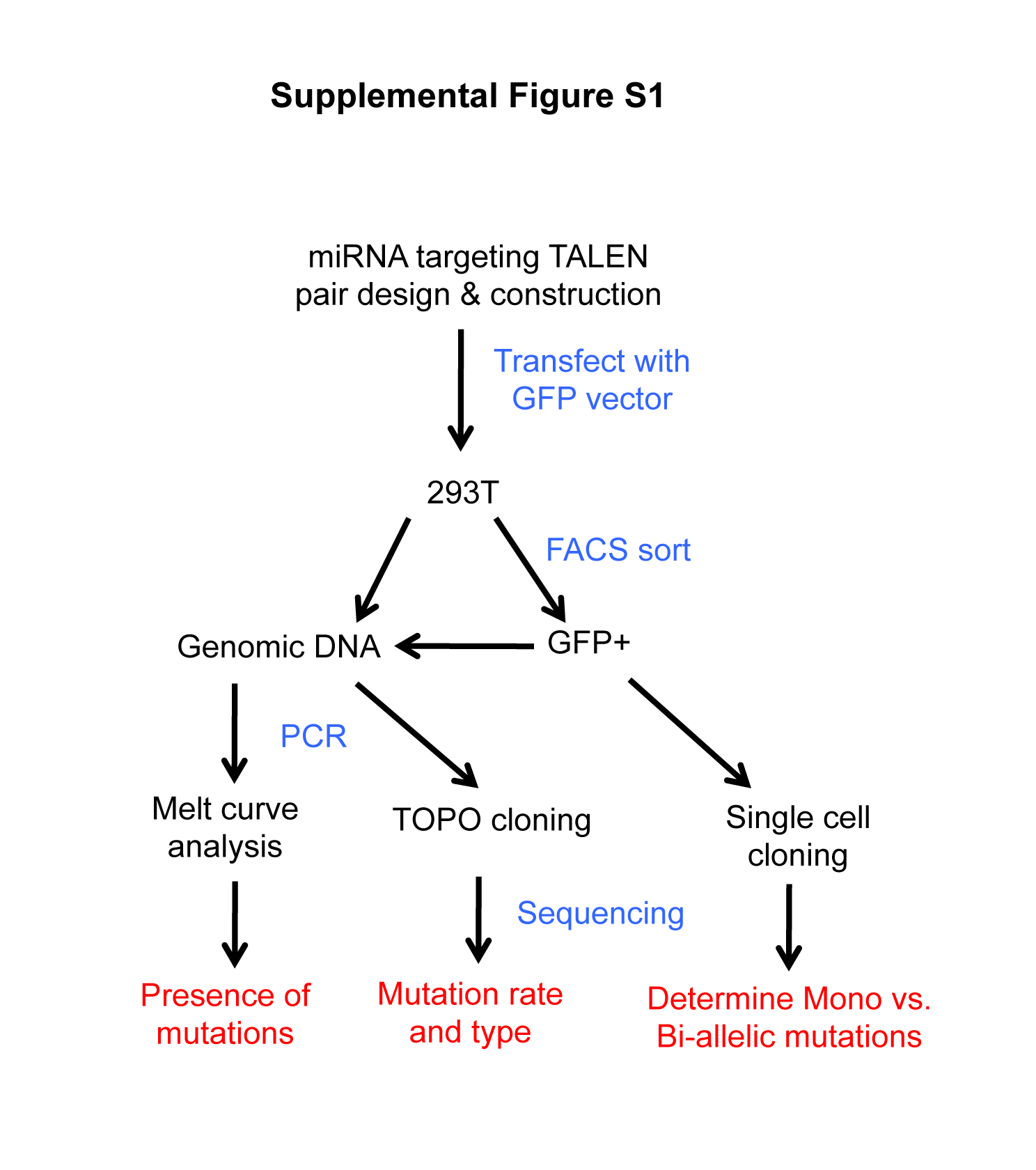

Supplement: Figure S1 — Experimental plan used to develop miRNA-targeting TALENs. TALEN pairs targeting different miRNAs were designed, constructed and transfected into 293T cells along with a GFP expression vector. After transfection, 293T cells were subjected to FACS sort to isolate cells with the TALEN pairs. gDNA was extracted from Wt, unsorted, GFP- or GFP+ cells. The TALEN pair-targeted regions were amplified by PCR and subjected to HRMA or TOPO cloning and sequencing to determine the presence of mutations. Moreover, GFP+ cells were plated in 96 well plates to obtain single cell clones. Single cell clones were subjected to PCR and TOPO cloning analyses to determine if bi- or mono-allelic mutations were being generated within the TALEN targeted regions. (TIF) [file pone.0063074.s001.tif]
